# Supplementary material for: Conservation Practices for Personal Protective Equipment: A Systematic Review with Focus on Lower-Income Countries
Source: Int J Environ Res Public Health. 2023 Jan 31;20(3):2575. doi: 10.3390/ijerph20032575 (PMC9915410; doi:10.3390/ijerph20032575)
Supplement: Supplementary file 1 [file ijerph-20-02575-s001.zip › ijerph-2073047-SM.pdf]

Supplemental Information

Contents

Search Terms – June 9, 2020 2

Inclusion & Exclusion Criteria for Title, Abstract, & Full Text Screenings 2

Inclusion criteria: 2

Exclusion criteria: 2

Extraction Form 3

General Information 3

Study Information 4

These questions should be answered for any studies that were RANDOM control trials. 5

Observational These questions should be answered for any OBSERVATIONAL studies. 6

Results 6

Primary Outcome (sometimes referred to as End Point) 6

Secondary (Endpoint) Outcome 6

Tertiary/additional Outcome (end point) 7

Other Outcomes (for clinical studies). This section is likely only applicable to studies in an applied, clinical setting (not the lab-based studies) 7

Summary of Each Disinfection Method in Included Disinfection Studies 9

Energetic Treatment 9

Ultraviolet Germicidal Irradiation (UVGI) 9

Sunlight and Microwave Irradiation 9

Dry Heat 9

Moisture and Heat 9

Chemical Treatment 10

Bleach 10

Hydrogen peroxide 10

Ethylene oxide 10

Mixed oxidants 10

Alcohol 10

Dimethyldioxirane 11

Peracetic acid 11

Risk of Bias and Quality Assessments 11

Search Terms – June 9, 2020

"personal protective equipment"[MeSH Terms] OR "personal protective equipment"[tiab] OR ("mask s"[tiab] OR "masked"[tiab] OR "masking"[tiab] OR "masks"[MeSH Terms] OR "masks"[All Fields]) OR ("protective clothing"[MeSH Terms] OR ("protective"[tiab] AND "clothing"[All Fields]) OR "protective clothing"[All Fields]) OR ("space suits"[MeSH Terms] OR ("space"[tiab] AND "suits"[All Fields]) OR "space suits"[All Fields]) OR ("respiratory protective devices"[MeSH Terms] OR ("respiratory"[tiab] AND "protective"[tiab] AND "devices"[All Fields]) OR "respiratory protective devices"[All Fields]) OR ("gowned"[tiab] OR "gowning"[tiab] OR "gowns"[All Fields]) OR ("N95"[tiab] AND ("mask s"[tiab] OR "masked"[tiab] OR "masking"[tiab] OR "masks"[MeSH Terms] OR "masks"[All Fields])) AND (“recyclability"[tiab] OR "recyclable"[tiab] OR "recyclables"[tiab] OR "recyclate"[tiab] OR "recyclates"[tiab] OR "recycler"[tiab] OR "recyclers"[tiab] OR "recycles"[tiab] OR "recycling"[MeSH Terms] OR "recycling"[tiab] OR "recycle"[tiab] OR "recycled"[tiab] OR "recyclings"[tiab] OR "reuse"[tiab] OR "reused"[tiab] OR "reuses"[tiab] OR "reusing"[tiab] OR "conservancies"[tiab] OR "conservancy"[tiab] OR "conservancy s"[tiab] OR "conservation"[tiab] OR "conservational"[tiab] OR "conservations"[tiab] OR "conservative"[tiab] OR "conservatively"[tiab] OR "conservatives"[tiab] OR "conserve"[tiab] OR "conserved"[tiab] OR "conserves"[tiab] OR "conserving”[tiab])

Inclusion & Exclusion Criteria for Title, Abstract, & Full Text Screenings

Inclusion criteria:

- Describes measures to conserve, reuse PPE
- Specifically addresses masks, gowns and/or face shields
- Includes an evaluative component
- Describes a process that addresses limited availability of PPE
- Takes place in any clinical healthcare setting

Exclusion criteria:

- Discusses only the “science” behind reuse (e.g. disinfecting) without including a discussion of the process of implementation
- Focuses on the conservation of medical equipment or devices
- Takes place in research or other non-clinical settings

HDI Calculations

In order to compare the applicability and global relevance of findings, we used the 2019 United Nations Human Development Index to stratify study settings by relative country development level. The HDI is a summary measure of achievement in health, education, and standard of living. Countries are considered to have a “very high” level of development with HDI of 0.800 or greater; “high” development with HDI of 0.700-0.799; “medium” development with HDI 0.550-0.699; and “low” with HDI < 0.550.

Extraction Form

The entire research team iteratively built and tested a data extraction form that accounted for the following components of included studies: background information (country, funding, etc.); type of PPE under study and its conservation strategy (e.g. reusing a disposable product, disposing of a product as instructed, etc.); approach used to disinfect PPE, if applicable; type of study (clinical vs. lab-based); type of disease and route of transmission for which PPE was being studied, if applicable; primary/secondary/tertiary end-points and results; study limitations and burdens.

General Information

Directions:
Log in to Covidence.
In the "Extraction" box, click on the link in the upper right corner labeled "XX studies to review".
Pick a study that does NOT have a "reviewer 1" or "reviewer 2" assigned.
Click "Start" under 'reviewer 1' or 'reviewer 2' in that grey box.
Click the back button to return to the previous page, where you can access the PDF version of the paper.
Return to this page and 'extract' all the data.
Return to Covidence and mark 'completed' so you know which manuscripts you've extracted. If you have an issue and need to remove your name from a reviewer slot, you can do this by clicking on the "manage reviewers" link below the grey box.
* Required

1. Name of Data Extractor
2. Covidence ID *
3. First Author Last Name
4. Title of Publication
5. Funding Source
    - University
    - Federal
    - Other grant
    - Private
    - Other:
6. Study Location (Country)

Study Information

7. Type of PPE Under Study [select all that apply]
    - Masks (surgical)
    - N95/respirators/elastomeric/HEPA
    - Gowns
    - Eyewear (shields, goggles)
    - Gloves
    - Other:
8. Sample size (number). [leave blank if not applicable or not listed]
9. Characteristics of PPE Under Study [select all that apply]
    - Involves disposable PPE under intended use
    - Involves reusable PPE (made to BE reusable) &/or their decontamination Involves extended (or multiple) use of disposable PPE
    - Involves disinfection (reprocessing) of disposable PPE Novel PPE (not mass produced, potentially homemade)
10. Comparator PPE, if applicable (leave blank if PPE above is not being compared to any other type of PPE) [select all that apply]
    - Masks (surgical)
    - N95/respirators/elastomeric/HEPA
    - Gowns
    - Eyewear (shields, goggles)
    - Gloves
    - Other:
11. Characteristics of Comparator PPE (if applicable) [select all that apply]
    - Involves disposable PPE under intended use
    - Involves reusable PPE (made to BE reusable) &/or their decontamination Involves extended (or multiple) use of disposable PPE
    - Involves disinfection (reprocessing) of disposable PPE Novel PPE (not mass produced, potentially homemade
12. Sample size (number) of comparator group. [leave blank if not applicable or not listed]
13. Type of Study
    - Lab
    - In-Situ (Clinical)
    - Other
14. Study Setting (Clinical) - Leave blank if answer to previous question was "lab"
    - Hospital inpatient
    - Outpatient setting
    - Disaster setting (ex: ebola response in Sierra Leone)
    - Other:
15. Type of Disease [select all that apply]
    - SARS-CoV-2 (Covid 19)
    - H1N1 (swine flu)
    - General influenza

- General viral/respiratory
- Bacterial
- Non-viral/bacterial (ex: chemicals)
- Not disease specific (ex: fit testing or efficacy of a mask)
- Other:

16. Disinfection study?
    - yes
    - no
17. If yes above: type of disinfection?
18. Type of Study
    - Observational
    - RCT
    - Other:

These questions should be answered for any studies that were RANDOM control trials.

19. How is it blinded?
    - No blinding
    - Double-blind
    - Single-blind (to study participants)
    - Single-blind (to experimenters)
20. What was the randomization approach?
    - Individual
    - Cluster
    - Other:
21. Does the study state the baseline characteristics of randomized and control groups?
    - yes
    - no
22. If listed, what are the baseline characteristics (names of demographic categories) of the groups? (if no characteristics, leave blank); 1) list in order of appearance in table if table exists, or 2) if not, in order listed in paragraph. Separate each characteristic with semicolon [list categories, avoid numbers]
23. If study states baseline characteristics, were any of the above listed baseline characteristics statistically significantly different (as indicated by $p<0.05$ or 95% CI including 0.0)?
    - yes
    - no
    - not specified
24. If Yes to above, which characteristics? (list in order of appearance in table if table exists, or if not, in order listed in paragraph. separate each characteristic with semicolon)

Observational These questions should be answered for any OBSERVATIONAL studies.

25. Type of Observational Study. *Mark only one oval.*
    - descriptive studies - Case Report (1 patient) descriptive studies - Case Series (multiple patients) Cross Sectional (snapshot in time)
    - Case Control (retrospective)
    - Cohort (groups of people with a common condition or exposure, can be prospective/longitudinal or retrospective)

Results

26. Length of main study/intervention/observation period
27. Length of final additional follow-up (after the study period), if applicable? [value; include units]
28. Attrition rate at final follow-up time point, if applicable? [value; include units}

Primary Outcome (sometimes referred to as End Point)

29. What was the primary outcome metric/variable?
    - infection rate (number, percent)
    - viral load
    - filtration factor
    - Other:
30. Result for PPE under study at end of main study/intervention/observation period end-point? [value; include units]
31. Result for PPE under study at final follow-up (after study period), if applicable? [value; include units]
32. Was the result for PPE under study significantly different than control (e.g. p value<0.05 or 95% CI ≠ 0)?
    - yes, outcome metric was higher than control
    - yes, outcome metric was lower than control
    - no
    - unspecified
33. Was this result significant at final follow-up (e.g. p value <0.05 or 95% CI ≠ 0)?
    - Yes
    - No

Secondary (Endpoint) Outcome

34. What was the secondary outcome metric/variable? (if available)
    - infection rate (number, percent)
    - viral load
    - filtration factor

- Other:

35. Result for PPE under study at end of main study/intervention/observation period end-point? [value; include units]
36. Result for PPE under study at final follow-up (after study period), if applicable? [value; include units]
37. Was the result for PPE under study significantly different than control (e.g. p value<0.05 or 95% CI ≠ 0)?
    - yes, outcome metric was higher than control
    - yes, outcome metric was lower than control
    - no
    - unspecified
38. Was this result significant at final follow-up (e.g. p value <0.05 or 95% CI ≠ 0)?
    - Yes
    - No

Tertiary/additional Outcome (end point)

39. What is the additional outcome metric/variable? (if available) *Mark only one oval.*
    - infection rate (number, percent)
    - viral load
    - filtration factor
    - Other:
40. Result for PPE under study at end of main study/intervention/observation period end-point? [value; include units]
41. Result for PPE under study at final follow-up (after study period), if applicable? [value; include units]
42. Was the result for PPE under study significantly different than control (e.g. p value<0.05 or 95% CI ≠ 0)?
    - yes, outcome metric was higher than control
    - yes, outcome metric was lower than control
    - no
    - unspecified
43. Was this result significant at final follow-up (e.g. p value <0.05 or 95% CI ≠ 0)?
    - Yes
    - No

Other Outcomes (for clinical studies). This section is likely only applicable to studies in an applied, clinical setting (not the lab-based studies)

44. Which potential biases or study design limitations are listed in the paper (example: selection, information, recall, and report bias) and confounding factors? [copy limitations section or copy paragraph here]

45. For studies where PPE has been used multiple times: Number of fit testing failures (for masks and respirators) after how many uses, over what period of time (ex: # failures / # uses or # failures / week); leave blank if not applicable or no info included
46. For fit test failures, was this result significant (e.g. p value <0.05 or 95% CI ≠ 0)? (leave blank if applicable)
    - Yes
    - No
    - Not specified
47. For all studies: Comfort level of users (list values and units) - leave blank if not applicable or no info included
48. Costs
    - Time/effort
    - Other:
49. based on your response above, what were the conclusions on the burdens or outcomes associated with the intervention. [1) copy and paste a table, 2) copy and paste the paragraph text, or leave blank if not applicable or not included]
50. List any references that might be relevant to our study

Summary of Each Disinfection Method in Included Disinfection Studies

Energetic Treatment

Ultraviolet Germicidal Irradiation (UVGI)

UVGI was determined to successfully decontaminate N95/filtering facepiece respirators but was not effective for eliminating influenza A and may increase filter penetrance by degrading respirator fibers. Because effective ultraviolet decontamination requires a minimum dose of UV to transmit to all porous layers of respirators, it carries the risk of material degradation; the extent of strength or efficacy loss may vary depending on the model of N95. Pulsed xenon ultraviolet treatment was studied for eyewear, glass carriers, and gown material, but could not be assumed effective and still required careful doffing and disposal after treatment.

Sunlight and Microwave Irradiation

High-energy radiation in the form of direct microwave irradiation melted parts of FFRs, making them unwearable for reuse. Masks could be modified so that lower-energy radiation may be sufficient for decontamination. According to Zhong et al., adding a layer of graphene to surgical masks enabled "self-cleaning" when exposed to solar illumination by reaching temperatures sufficient for viral denaturation and inactivation.

Dry Heat

3M respirators could be sterilized for multiple reuses using dry heat without altering their efficacy but in another study of respirators, dry heat did not result in sufficient reduction of inoculated organisms, suggesting that moisture is essential for inactivation of contaminants on masks or respirators.

Moisture and Heat

Steam treatment effectively decontaminated face masks and respirators from Escherichia virus MS2, a single-stranded RNA bacteriophage that targets E. coli; methicillin-resistant staphylococcus aureus (MRSA); and avian influenza avirus. It could enable reuse for up to 10 days without a reduction in filtration factor. Steam sterilization made 3M-brand masks safe for reuse but results may be model-specific. Although Liao et al. found that vapors may damage filters, other studies found that moist heat incubation was effective in eliminating viral contaminants without reducing filtration efficacy. Microwave-generated steam (MGS) effectively decontaminated respirators for multi-donning reuse without degradation of efficacy (multi-donning fit factor) or comfort. Microwave steam bags inactivated MS2 with 99.9% efficacy but sporadically left viable H1N1 virus; thus, further studies are required before endorsing MGS decontamination for reuse.

Chemical Treatment

Bleach

Bleach effectively disinfected respirators, half-mask elastomeric respirators, gowns and gloves but can also leave strong odors, discoloration, and corrosion of metal and may degrade respirator filters. Elastomeric respirators designed to be reused were found to withstand up to 45 days of reuse after daily disinfection using a Standard Operating Procedure for adequate disinfection with a bleach solution. Using bleach with detergent was effective for removing influenza H1N1 from elastomeric respirators. Lawrence et al. measured the effectiveness of repeated cleaning with detergent with and without bleach disinfection on reusable half-mask elastomeric respirators (HMERs) and powered air-purifying respirators (PAPRs) experimentally contaminated with H1N1 influenza. Both HMERs and PAPRs had a significant log reduction in influenza with the detergent alone, the efficacy of which that was not significantly increased with the addition of bleach disinfection. The viral load of a surrogate virus was effectively reduced when gloves and gowns were decontaminated using a bleach wipe and/or spray. However, "germicidal wipes" were ineffective for removing contaminants from reusable eyewear. This study evaluated bacterial contamination of surgical eye protection after disinfection with germicidal wipes and found high rates of bacterial contaminants after disinfection. Because of these findings, the author advised against reusing eye protection.

Hydrogen peroxide

Vaporized hydrogen peroxide was effective in treating respirators and prolonging use and was feasibly implemented on a large hospital-wide scale. Both liquid and vaporized hydrogen peroxide did not leave residue on FFRs after treatment. Studying a surrogate comparable to SARS-CoV-2, Cheng et al. found that spraying ionized hydrogen peroxide could inactivate influenza A virus contaminants from respirators.

Ethylene oxide

Ethylene oxide effectively treated respirators without changing filter aerosol penetration and resistance. However, it was observed that gaseous ethylene oxide treatment may have left toxic residues.

Mixed oxidants

Mixed oxidants (10% Oxone, 6%, sodium chloride, 5% sodium bicarbonate) could disinfect FFRs but left odors and oxidation on metal components of respirators. In another study, oxygen-based disinfectants were not considered effective, and only two of ten commercially available disinfectants (one with 2% chlorine and the other 1.75% peracetic acid), effectively inactivated spores.

Alcohol

Wipes with 70% isopropyl alcohol did not eliminate H1N1 virus, but a combination of quaternary ammonium chloride with isopropyl alcohol was effective in disinfecting reusable elastomeric respirators. However, liquid alcohol disinfectant may present the risk of degrading respirator filters.

Dimethyldioxirane

Dimethyldioxirane caused odors and metal oxidation of FFRs.

Peracetic acid

2% peracetic acid effectively inactivated bacterial spores. A thin layer of combined PAA with detergent was effective for decontaminating hydrophobic PPE surfaces, but submersion or covering with only PAA was ineffective.

Risk of Bias and Quality Assessments

SI Table 1. Risk of bias assessment of included cohort & case-control studies

	**Exposed Cohort Repre-sentativeness**	**Non-exposed cohort selection**	**Ascertainment of exposure**	**Absence of outcome at start**	**Comparability of cohorts**	**Assessment of outcome**	**Length of follow-up**	**Adequacy of follow-up**
Bessesen (2015)	1	1	1	1	0	0	N/A	N/A
Ang (2010)	1	1	1	1	0	1	N/A	N/A
Duarte (2010)	1	N/A	0	0	0	0	N/A	N/A

	**Case definition**	**Case representativeness**	**Selection of controls**	**Definition of controls**	**Comparability of Cases and Controls**	**Ascertainment of Exposure**	**Method of Ascertainment**	**Non-Response Rate**
Wang (2020)	1	1	0	0	0	1	1	0

*Score of 0 indicates risk of bias; a score of 1 or 2 was awarded if the study took appropriate measures to limit the risk of bias.

	Random sequence allocation	Allocation Concealment	Selective Reporting	Blinding (participants and personnel)	Blinding (outcome assessment)	Incomplete outcome data
Viscusi 2011 [59]	+	?	?	+	?	?
MacIntyre 2015 [64]	+	−	?	−	+	+
MacIntyre 2013 [49]	+	−	+	−	+	+
Radonovich 2019 [70]	+	−	+	−	+	+
Jacobs 2009 [67]	+	−	+	−	?	+
MacIntyre 2011 [63]	+	?	?	−	?	+
MacIntyre 2014 [71]	+	?	?	−	?	+
Au 2010 [74]	+	−	?	−	?	?
Cowling 2008 [68]	+	−	?	−	?	+
Loeb 2009 [66]	+	?	?	−	+	+
Mukerji 2017 [65]	+	?	?	−	?	+

SI Figure 1. Risk of Bias for Included RCTs
